# Supplementary material for: Are carbon emissions trading and green financial instruments synergistic? -Comprehensive quantitative research based on content analysis
Source: PLoS One. 2024 Mar 7;19(3):e0298601. doi: 10.1371/journal.pone.0298601 (PMC10919601; doi:10.1371/journal.pone.0298601)
Supplement: S1 Data — (ZIP) [file pone.0298601.s001.zip › Data/地级市Z_F工作报告.zip!/║■─╧/╧μ╠╢╩╨/└┤╫╘╣1⁄2╓┌║┼í╛┬φ┐╦╩2╛▌═°í┐_2019.docx]

各位代表：

现在，我代表市人民政府向大会报告工作，请予审议，并请各位政协委员和其他列席人员提出意见。

一、2018年工作回顾

2018年，市人民政府高举习近平新时代中国特色社会主义思想伟大旗帜，面对复杂形势，扛牢使命职责，迎难而上，奋进有为，朝着实现全面建成小康社会目标迈出了新的步伐。全年实现地区生产总值2161.4亿元，同比增长7.8%，固定资产投资增长10.1%，规模工业增加值增长7.4%，社会消费品零售总额增长10%，引进内资增长18.3%，利用外资增长12.2%，财政总收入增长6.6%，城乡居民人均可支配收入分别增长7.9%和8.5%，经济社会发展实现了稳中有进。一年来，主要做了以下工作：

（一）聚焦实体经济，加快产业转型升级。深入开展“产业项目建设年”活动，舍弗勒、湘钢技改等46个项目入选省“五个100”工程，吉利新能源SUV、桑顿新能源三期等项目竣工投产。持续发力三大千亿产业，装备制造业产值达834.2亿元，军民融合产业产值达420亿元，汽车产业产值达562.9亿元，“1+4”特色产业规模工业增加值占比达到71.6%。服务业对经济增长的贡献率达到48.7%，三次产业结构比调整为5.8：48.2：46.0。岳塘区入选全国绿色发展百强区，天易经开区跻身千亿园区行列，雨湖工业集中区转型为省级高新技术产业开发区。韶山红色旅游成功申报国家级服务业标准化示范项目，湘潭县、湘乡市、昭山示范区入围省精品旅游线路重点县。推行链长抓统筹、盟长促配套、行长帮融资的“三长”助推产业工作机制，推进省级小微企业金融服务试验区建设，开展中小微企业应收账款融资专项行动，全年新增产业信贷投放72亿元。实施企业上市“蝶变计划”，地通控股等10家重点企业上市步伐加快。加强产业项目用地保障，强化闲置土地、低效用地清理处置，完成用地报批1万余亩，实现土地经营收入103亿元。着力推进长株潭国家自主创新示范区建设，出台了“智造谷”产业发展规划，实施创新引领“四个10”重大科技专项，完成发明专利申请2800件，增长30%；新增高新技术企业67家，增长36%；完成高新技术产业增加值730.3亿元，增长12%。新引进“两院”院士5人，新增产学研创新团队10个。高校毕业生留潭率达15.2%，提高5个百分点。

（二）打好系列组合拳，三大攻坚战开局良好。全力防范和化解政府性债务风险，盘清了债务底数，制定了未来10年债务化解方案。政府性投资项目调减92.1%，市直“三公”经费支出下降17.8%，争取专项债券资金置换政府债务66.7亿元，通过公共预算安排、政府性资金调度、土地出让、资产处置等方式筹集化债资金约40亿元，牢牢守住不发生区域性风险的底线。构建“大环保”工作格局，完成第二次全国污染源普查入户调查和“三线一单”编制。积极配合中央环保督察“回头看”和省级环保督察，如期完成富力城项目涉绿整改，“绿心”地区54个违法违规建设项目、48个工业退出项目整改任务全面完成，进度领先长株潭三市，成效获得国省好评。统筹实施工业污染防治“飓风行动”、扬尘污染治理“亮剑行动”、打击非法挖山采砂采石“利剑行动”，开展工业企业排放、工地扬尘、餐饮油烟、秸秆焚烧、汽车尾气等专项治理。湘钢启动实施大气污染防治十大工程，烟气脱硫、防风网建设、转炉湿改干等环保治理项目全面完工。全年空气质量完成省定“三降一升”目标，优良率为76.6%，提高3个百分点。深入落实河长制，编制“一河一策”，实施涟水流域污染专项治理。加强城区污水管网及乡镇污水处理设施建设，共完成32条黑臭水体基础治理，6个集中式饮用水源地水质均达到或优于Ⅲ类，水质达标率100%。病死畜禽无害化处理中心和收集转运体系建成投入使用。湘乡市生活垃圾焚烧发电项目竣工。竹埠港重金属治理项目、牛头化工一期土壤治理项目全面完工，南天化工等土壤污染治理稳步推进。锰矿地质环境治理示范工程基本完成，成功申报谭家山矿区生态修复项目。全年办理环境违法案件218起，刑事拘留21人，形成了强大震慑。开展脱贫攻坚“三落实”集中行动，全市3.2万多名党员干部与贫困户结对帮扶，成立扶贫产业合作组织185个，带动1万多贫困户脱贫致富，解决了1.06万贫困人口农村饮水安全问题。在全省率先运行健康扶贫市级“一站式”综合结算平台，实现“扶贫特惠保”等医保政策“四个全覆盖”。梅桥、中沙易地扶贫搬迁安置点投入使用。全年脱贫21742人，基本实现除兜底保障外的贫困人口脱贫，贫困发生率降至0.36%。对口援助吐鲁番市高昌区成效显著，对口帮扶永顺县“两产两带”经验在全省推介。奋斗在防范风险最前线、拼搏在环保治理最前沿、奔走在精准扶贫最前方的干部群众，用心血奠定了打好三大攻坚战的坚实基础，用付出谱写了感人肺腑的奉献之歌。

（三）以供给侧结构性改革为主线，全面深化改革开放。深化“放管服”改革，公布市本级“马上办、网上办、就近办、一次办”事项1073项，市本级3251项政务服务事项纳入“互联网+政务服务”一体化平台运行，依申请类政务服务事项网上可办率达97%。韶山旅游“先行赔付”成为全省“放管服”改革亮点工作。协同推进“多证合一”、证照联办，大力推广电子营业执照应用，新增各类市场主体2.7万户。驻潭央企、省企“三供一业”分离工作进度和成效走在全省前列。注实投融资公司资产，以地产集团先行先试，统筹推进平台公司减员增效、“强身健体”和市场化转型。市政公司改制组建为市政集团。全面完成市级公共资源交易平台整合改革任务。推进增量配电改革，获颁全省首张供电类业务许可证。积极参与首届中国国际进口博览会等重大招商经贸活动，成功举办首届潭商大会、第七届湖南省电子商务大会、第十三届中国湘菜美食文化节。聘请了首批招商顾问，分别设立京、沪、粤驻外招商联络处。新签约亿元以上项目106个，引进“三类500强”项目18个，吉利新能源跨界车获引进“500强”省级优秀项目，京东、阿里巴巴菜鸟物流、中节能废矿物油再生项目、昭山国际知创文化基地等项目签约落地，岳塘国际商贸城一期开业，京东电商产业园顺利开园，恒大养生谷四大园建成开放，北大未名生物医学创新示范园、湖南惠景生殖与遗传专科医院等项目进展顺利。湘潭成为全省第一个全国供应链创新与应用试点城市，步步高获批为试点企业。在全省开放型经济引领能力排行榜中我市位居第三。

（四）科学铺排项目，稳步提升城市品质。启动新一轮城市总规编制前期工作，开展湘潭经开区等6个重点发展片区的控制性详规编制，完成城区中小学、幼儿园、文化体育、医疗卫生设施等9个专项规划，完成城市核心区“一江两岸”、竹埠港、碧泉湖、九华湖等6个城市设计。应对融资形势变化，合理调整城建项目盘子，重点保障和推进基础设施补短板。完成湘江两岸防洪带建设13.7公里，拉通了新马路、晓塘路、白云路等城市干道，启动了荷塘客运枢纽、河西公共客运总站等交通枢纽建设，一大桥交通优化及综合整治项目建成通车，昭华大桥即将通车，杨梅洲大桥、下摄司大桥建设稳步推进。开工“双修双改”项目638个，提质改造小街小巷20条，完成了103个社区、85个村的市容专项整治。海绵城市、地下综合管廊建设有序推进。窑湾历史文化街区一期开街运营，取得了打造旅游热点、做旺景区人气的初步效果。湘潭大学南校门广场改造等项目顺利完成，以崭新的面貌迎接了60周年校庆。完成55个小区增绿补绿，仰天湖、百亩湖等公园建成开放，城市建成区绿化覆盖率达43%，成功获批国家森林城市。坚持“房住不炒”定位，积极稳房价、稳地价、稳预期，房地产市场保持健康平稳。新开工棚户区改造1.7万户，分配公租房1121套，全省首个房屋租赁综合服务平台上线运行。各县市积极提升城市品质，湘潭县荣获省级卫生县城、省级园林城市，韶山市成为全国人居环境整治先进市。莲城大地一幅风生水起、方兴未艾的城乡画卷跃然如见，前景可待。

（五）加强统筹谋划，大力推进乡村振兴。完成全市乡村振兴总体规划，明确了乡村振兴的总体目标、重点任务和推进路径。10大现代农业示范园引入新型经营主体127家。湘莲、槟榔、肉类加工等特色产业升级明显，优质粮油工程稳步实施，非洲猪瘟等农业风险有效防控，农业综合效益稳步提升。实施土地综合整治8.7万亩，完成种植结构调整21万亩。推进农村集体产权制度改革，基本完成清产核资主体任务，土地确权登记颁证合同签订率达98%以上，耕地流转率达60.5%。韶山市获批全国农村集体产权制度改革试点。完成水利薄弱环节综合治理项目35个，修复较大水毁水损设施1500多处。积极应对近年少有的旱情，确保全市用水安全，韶山灌区成功创建国家水情教育基地。提前1年完成贫困村电网改造，农村户均容量位居全省第二，湘乡市、韶山市列入全国首批小康用电示范县。完成自然村通水泥（沥青）路建设135公里，提质改造农村公路215公里，新增农村公路会车平台1500处，完成生命安全防护工程376公里，韶山市获“四好农村路”省级示范县。韶乌线一期等城际道路建成通车。水府大桥正式通车，库区群众“出行难”问题得到根本改善。启动实施农村人居环境整治三年行动计划，完成448个行政村村庄规划编制，打造省级美丽乡村示范村20个，完成“一江两水”沿岸厕所革命2.4万座，破败空心房整治有序推进,改造农村危房3310户。重点打造盘龙生态小镇、花石湘莲小镇、水府水乡小镇、平里旅游小镇。办好了首届中国农民丰收节湖南主会场活动。湘乡市获全国农作物生产机械化示范市。湘潭县获全国农村创新创业典型示范县。

（六）坚持民生优先，不断增进人民福祉。全年城镇新增就业6.3万人，失业人员再就业2.4万人，城镇登记失业率控制在3.5%以下。居民消费价格指数控制在3%以下。加快处置建设领域历史遗留问题，依法追缴税费等款项7亿余元，办理不动产权证1万多户，一批成因复杂、久拖不决、人民群众意见较为集中的遗留问题，得到了创造性解决。城乡居民医保人均补助、城乡低保、特困供养、残疾人补贴、老年人福利全线提标，保障水平位居全省前列。全市养老床位达到2万张。市殡仪馆改扩建项目竣工投入使用。湘钢一中十二中校区、和平小学科大校区完成主体建设，湘乡起凤学校、岳塘滴水湖小学建成招生，顺利回购益智中学，新增义务教育学位8481个，全部消除超大班额。新增普惠性民办幼儿园64所。进城务工人员随迁子女就学全面享受同城待遇。开展教材教辅、食堂管理、民培机构专项治理，解决了一批人民群众关切的教育热点难点问题。启动基层医疗卫生服务能力提升三年行动计划，临床重点专科能力和基层医疗卫生服务水平不断提升。深化公立医院管办分离、编制人事等改革，启动县乡村医疗卫生机构一体化改革，韶山市率先实现家庭医生签约全覆盖。组织了“我们的中国梦·文化进万家”等系列文化惠民活动，建成24小时自助图书馆、图书馆分馆、流动图书点共200余个。“湘潭市少年儿童主题读书活动”成功创建国家公共文化服务体系示范项目。指导开展了毛泽东同志诞辰125周年、彭德怀同志诞辰120周年群众性纪念活动。成功举办第五届中国（湘潭）齐白石国际文化艺术节、韶山红色国际马拉松、昭山芒果音乐节等活动。积极组织参加省十三届运动会和残运会。湘潭广电参与拍摄的电视剧《彭德怀元帅》获飞天奖。肉菜追溯体系建设做法获商务部推介。“一桌餐”“私房菜”“打击非法会议营销”等专项治理成效显著。积极创建国家安全发展示范城市，安全生产形势稳定向好，事故数和死亡人数分别下降23.5%和11.3%，全年未发生较大及以上事故。健全立体化治安防控体系、多元化矛盾化解体系，强力开展扫黑除恶专项斗争，全市公众安全感测评排全省第一，韶山市排县（市）第一。配合做好了军队、武警部队改革，国防建设、民兵工作和人防发展取得新成效，民兵调整改革取得全国第三的好成绩。

（七）落实全面从严治党要求，政风行风持续好转。积极实施“党建领航”工程，认真落实“四责四诺”“五联五问”要求，大力推广一线工作法。办理群众信访事项7872件次，“四大攻坚”信访积案、中央第八巡视组交办件、省委第二巡视组交办的群众身边问题全部按程序办结。市长热线、市长信箱全年接收群众来信来电10万多件次，办结率达到98%。清理规范性文件674件，废止、失效、取消287件，修改10件。推动出台了《湘潭市城市绿化条例》等法规。办理行政复议案件145件，各人民调解组织参与化解社会矛盾纠纷1.1万件。在全省率先实现“一村（社区）一法律顾问”。推动廉洁政府建设，严格落实“一岗双责”，全面铺开领导干部自然资源资产离任（任中）审计。坚决拥护支持省委、省纪委监委对陈三新案、赵文彬案的处理决定，坚定不移推动全面从严治党向纵深发展。认真落实监察体制改革、税务系统改革等改革任务。坚持向人大及其常委会报告工作，向人民政协通报情况。认真听取民主党派、工商联、无党派人士及人民团体意见。全年共办理人大代表建议502件、政协提案329件，办理满意率达100%。

统计、民族宗教、妇女儿童、青少年、老龄、工会、科协、社科联、侨联、文联、残联、移民、气象、水文、国安、档案、地方志、住房公积金、供销合作、外事、侨务、对台经贸交流、港澳事务等工作实现了新进步，取得了新进展。

各位代表，2018年各项成绩的取得，最根本最重要的就在于有习近平新时代中国特色社会主义思想的科学指导，有以习近平同志为核心的党中央的正确领导；离不开省委、省政府和市委的坚强领导，得益于市人大、市政协和社会各界的支持监督，凝结了全市人民的努力奋斗。在此，我代表市人民政府，向全体人大代表、政协委员，向各民主党派、工商联、无党派人士、新社会阶层人士和各人民团体，向离退休老同志，向驻潭部队、武警官兵、政法干警和消防指战员，向所有关心支持湘潭改革发展的各级领导、各界朋友，表示衷心的感谢并致以崇高的敬意！

各位代表，一切过往，皆为序章。过去的一年，发展环境错综复杂，改革任务繁重艰巨，新旧动能深刻转换，下行压力持续加大。唯其艰难，才更显成绩的来之不易；唯其不易，才更显历程的弥足珍贵。

——这一年，我们坚决贯彻习近平经济思想，高质量发展呈现出蓬勃势头。全市税收占财政总收入占比达78.6%，提升6.7个百分点；非税收入占地方财政收入占比为36.4%，下降8.3个百分点。全部投资中民间投资占比达72.2%，产业投资中技改投资占比达36.3%，高新技术产业投资占比提高了4个百分点。全社会研发经费投入占GDP比重达2.2%，发明专利申请数、新增高新技术企业数、高新技术产业增加值等创新指标增速位居全省前列。质量变革、效率变革和动力变革不断深化，汇聚了百流归一、积蓄迸发的高质量发展伟力。

——这一年，我们始终把防范化解重大风险作为重中之重，稳住了大局、守住了底线。我们尽最大的努力取得了各界支持，调动了多种资源，坚定了各方信心，塑造了负责任、守信誉的政府形象。我们多措并举筹集化债资金，通过密集走访争取金融机构延期展期、认购债券、置换债务，确保了不断链、不爆雷，不发生重大舆情事件。我们钻坚研微啃政策，百计千方创模式，为下一步的攻坚克难奠定了基础、积累了经验、趟出了新路。我们迎着问题进，顶着困难上，闯险滩、避暗礁、排雷区，负重前进的步伐更加踏实、更为坚定。

——这一年，我们以配合做好国务院大督查、中央环保督察“回头看”、省委巡视和国省审计为契机，扎实抓好问题整改，统筹开展各项工作，全面推进创先争优。产业转型升级经验受到国务院第五次大督查通报表扬。“三长”助推产业发展机制被国务院大督查作为经验推介。连续8年获全省公共卫生服务工作先进，安全生产、计划生育、社会治安综合治理连续三年获全省先进，打非处非、粮食安全等7项工作绩效居全省第一，黑臭水体治理、食品药品安全监管等5项工作获得国省表彰或推介。跻身全国产业竞争力百强城市和城市品牌百强排行榜。所有这些成绩，是全市新时代奋斗者攻坚克难闯出来的、挥洒汗水拼出来的，更是全市上下撸起袖子干出来的。

——这一年，我们以改革开放再出发的坚定信念，着力打基础、利长远，发展活力进一步激发。政务审批改革、机构改革、投融资体制改革、国有企业改革、农村土地制度改革等各领域改革深入推进。创新推出重大产业项目投资企业承诺制，产业项目落地开工进度显著加速。始终把开放当作发展的主旋律，以积极的姿态“走出去”，以满腔的热忱“请进来”。腾讯、阿里等新经济纷至沓来，众泰、银帝等新业态接踵而至，更多的企业落户湘潭，潭商回潭成为热潮。在湘潭大地上，各类人才尽展其能，各种资源集聚汇合，激荡起奔流不息的创新创业浪潮。

——这一年，我们坚持以人民为中心的发展思想，既尽力而为又量力而行，保障和改善民生“不打折、不欠账”。坚决兑现承诺的民生实事，解决了一批人民群众最期盼、最有感的身边事。一批餐饮点的油烟排放得到有效治理，一批豆制品店、米粉店、炒货店纳入监管，一批老旧小区实现提质改造，更多的群众享受到了厕所革命、“四好农村路”、新生儿疾病筛查、妇女“两癌”检查等民生实事带来的政策实惠，感受到了党和政府的阳光温暖。民生支出占财政支出比重稳步提升，我们以干部的“辛苦指数”换来了人民群众的“幸福指数”，以政府的“紧日子”换来了人民群众的“好日子”。

各位代表，2018年的成绩，是在“三期叠加”的复杂环境和经济下行的现实压力下取得的，是在三大攻坚战特别是全力防范化解政府性债务风险的背景下取得的。在充分肯定成绩的同时，我们必须清醒地认识到，地区生产总值、固定资产投资、规模工业增加值、社会消费品零售总额4项指标增速未能达到年初预定目标。主要是国内外经济形势发生了一系列重大变化，宏观经济下行的巨大压力与湘潭自身产业转型升级、新旧动能转换、经济结构调整、防范化解政府性债务风险等各种因素交织影响，给实现全年目标增添了远超年初预期的较大变数。虽然上述指标未能达到目标，但相对于比较复杂的发展环境，我市各项经济指标增速仍然保持在合理区间，基本达到或超过全省平均水平，取得了现实条件下能够达到的较好结果。这些差距的显现，深化了我们对新形势下经济工作的规律性认识，有助于我们更好地把握高质量发展的特点，为我们做好2019年的经济工作提供了深刻的借鉴、积累了宝贵的经验。

同时，部分项目特别是基础设施项目受政策因素的影响，暂停或暂缓实施，建设进度不理想；新兴产业还未能形成有力支撑，“新不足以补旧、小不足以补大”的问题未能得到有效破解，经济可持续发展和财政可持续增收的基础不牢；经济增长还存在较大的投资依赖惯性，民间投资活力不足，产业投资占比有待继续提高；三大攻坚战任务艰巨，大气、水、土壤污染治理任务繁重，防范化解政府性债务风险压力巨大；实体经济比较困难，政策落地存在时滞效应，缓解融资难、融资贵的系列举措见效不快、效果不佳，营商环境尚需进一步改善；就业、教育、医疗等方面与群众期待还有差距，农村基础设施建设还有不少欠账；一些干部适应新形势、解决新问题的能力不强，不作为、慢作为甚至乱作为现象不同程度存在。这些问题，有的是长期以来制约湘潭发展的老问题，有的是随着时间推移涌现出来的新问题，务必引起我们的高度重视，采取切实有效措施加以解决。

二、2019年工作安排

2019年是新中国成立70周年，是决胜全面建成小康社会第一个百年奋斗目标的关键之年。我们将以习近平新时代中国特色社会主义思想为指导，创造性贯彻落实中央、省委和市委经济工作会议精神，坚持稳中求进工作总基调，坚持新发展理念，坚持推进高质量发展，坚持以供给侧结构性改革为主线，坚持深化市场化改革、扩大高水平开放，统筹推进稳增长、促改革、调结构、惠民生、防风险工作，进一步稳就业、稳金融、稳外贸、稳外资、稳投资、稳预期，大力实施“三大攻坚、千百扶培、招大引强、双十提升、惠民暖心”五大行动，保持经济持续健康发展和社会大局稳定，为全面建成小康社会收官打下决定性基础，以优异成绩迎接中华人民共和国成立70周年。

各位代表，2019年，危和机同生并存的态势将更为明显，我们处在一个愈进愈难、愈进愈险，而又不进则退、非进不可的关键时期。我们要准确把握稳中有变、变中有忧的形势，辩证看待内外条件发生的巨大变化，增强忧患意识，坚定必胜信心，把握主动，善用优势，提升推进湘潭高质量发展的五大能力。

一是增强产业竞争力。坚决落实中央、省委经济工作会议精神，把握政策取向，用好政策红利。继续开展“产业项目建设年”活动，围绕打造“智造谷”和汽车城、军工城、文创城“一谷三城”，部署产业链、创新链、资金链，集聚资本流、技术流、人才流，推进产业聚合发展。支持骨干企业做强做优，推进优质企业“蝶变”上市，帮助中小企业转型升级，鼓励本地企业上下游配套，全面落实支持民营企业高质量发展系列政策，使产业成为防范风险的“压舱石”、稳定增长的“驱动器”、区域发展的“强引擎”。

二是培育创新驱动力。持续推进以科技创新为核心的全面创新，突出企业主体和产学研用结合，确保全社会研发经费支出占GDP比重达2.4%以上。积极推进符合新形势、新要求的投融资、项目建设、人才引进、土地开发、国有企业经营管理等机制创新，对标高质量发展指标，着眼集约高效，努力提高投资、土地、人才等要素的产出效率。采取有效措施，切实增强各类平台发展动力，充分发挥湘潭高新区、经开区、昭山示范区等产业园区发展活力，积极推进地产、城发、产业等国有企业转型发展。

三是提升区域融合力。全力抢抓长株潭融城机遇，主动对接长沙南部片区开发建设，积极谋划发展、规划产业、安排项目，坚定不移推进“湘潭向北”城市发展战略。盯紧基础设施补短板机遇，对接城际交通、物流、市政基础设施、公共服务设施等重点领域铺排项目。坚决落实省委、省政府统一部署和长株潭三市共同行动计划，更加积极地推动解决一体化发展中的难点、堵点问题。努力在一体化中找准定位、放大优势，培育核心竞争力，进一步展现湘潭作为、提升湘潭地位、增强湘潭能级。

四是强化风险防控力。盯紧环境污染、社会治安、安全生产、信访维稳、涉众金融等风险点，切实加强对企业经营、房地产市场、对外贸易、城乡就业等重点领域的预研预判，全力维护改革发展稳定大局。坚持以“不发生区域性系统性金融风险”为底线，全力推进债务平滑基金、国寿基金、片区合作开发等政府性债务化解新模式，努力实现“债务结构更加合理、融资方式更加多元、资金使用更加高效”的目标。统筹防风险和稳增长的关系，始终把发展作为化债务、防风险的根本出路。

五是打造环境软实力。以增强微观主体活力为重点，推动相关改革走深走实。聚焦群众和企业需求，坚持把优质服务群众、有效保障企业作为政府工作的努力方向。全面释放机构改革红利，以“放管服”的“硬措施”，增强营商环境的“软实力”。完成市级政务信息资源共享和“互联网+政务服务”一体化平台建设，实现70%以上服务事项“一窗”受理、网上可办。努力以最少的审批项目、最高的办事效率、最好的政务服务，让企业和群众花最少的时间，跑最少的路，有最满意的获得感。

根据中央、省委和市委经济工作会议精神，建议今年全市经济社会发展主要预期目标为：地区生产总值增长8%左右，固定资产投资增长10%以上，规模工业增加值增长7%以上，财政总收入增长6.5%以上，进出口总额增长15％以上，社会消费品零售总额增长10％左右，城镇登记失业率控制在4%以内，居民消费价格指数涨幅控制在3％以内，城乡居民人均可支配收入与经济增长同步，万元GDP综合能耗下降3.6%以上。

围绕以上目标和要求，主要做好十个方面的工作：

（一）着力推动特色产业高质量发展。抢抓5G商用机遇，加快人工智能、工业互联网等新型基础设施建设，落实“智造谷”产业发展规划及三年行动计划。深入推进三大千亿产业发展，力争装备制造产业稳定在千亿元规模，汽车产业产值达700亿元以上，军民融合产业实现产值500亿元左右。完善产业链和产业联盟工作机制，组建产业链专家咨询委员会，发挥好“三长”联动工作机制作用。全年完成产业项目投资800亿元以上，产业投资占固定资产投资比重达50%以上。深化国防科技工业改革，加快军民融合产业发展，积极创建长株潭军民融合创新示范区。推进吉利新能源跨界车、通瑞汽车、蓝思科技、舍弗勒等一批重大产业项目，加快雨湖产业新城、湘乡新能源汽车产业园等项目建设，打造“135”工程升级示范样板。积极壮大步步高、岳塘国际商贸城等骨干企业和重点项目，着力打造湖南商贸物流核心枢纽。支持韶山、昭山深化全国全域旅游发展示范区创建，抓好窑湾历史文化街区开发运营，支持盘龙大观园创成5A级景区，加快乌石旅游区创5A进度，推进“最忆韶山冲”等文化旅游项目建设。开展“千户中小企业精准帮扶行动”，建立信息服务平台，完善融资担保机制，深化小微企业金融服务试验区建设，支持湘潭金融商务区发展。深化企业上市“蝶变计划”，努力实现企业上市新突破。实施机器人产业链创新培育等10大自主创新示范工程，推进新能源汽车电控驱动等10大科技成果转化项目，抓好车用高性能锂电池开发等10大科技创新团队建设，建设运营好华研实验室等10大科技创新平台，力争全市高新技术企业达到300家以上，产业增加值达800亿元以上，高新技术产业投资占产业投资比重为18%左右。深化全国质量强市示范城市创建，打造湘潭质量品牌。

（二）着力做强园区经济和县域经济。支持园区各项改革，帮助园区切实解决面临的困难和问题，让园区坚守发展产业的初心，发挥政策环境的优势，防止过度“行政化”趋向，始终担当好全市经济建设主战场的使命。支持湘潭高新区建设“智造谷”核心区，综合实力迈向国家级高新区前列。支持湘潭经开区以打造“汽车城”、建设“湘江湾”为目标，全面发挥产业主阵地、融城桥头堡作用。支持昭山示范区优化产业布局，加快与长沙南部片区的全面融合。支持天易经开区对标国家级经开区，全面完成培育期各项指标任务。支持湘乡经开区调区扩区，迈进省级经开区20强。支持韶山高新区主攻食品、新材料及高端制造业，实现发展进位。支持岳塘经开区以岳塘国际商贸城和竹埠港新区为引擎，加快打造全省商贸强区。支持雨湖高新区抢抓融城机遇，加快华夏幸福产业新城建设。鼓励各县市对标全国百强县榜单和全省经济强县名单，努力实现争先进位。支持市县两级平台公司、国有企业和政府性担保机构相互参股，探索以“结对子”“一区多园”等方式，推动市县两级产业园区携手发展。大力推进新型城镇化试点，坚持和完善推进乡镇经济上台阶的系列奖励政策，加大对中心镇的政策、资金、项目支持力度，着力打造一批具有较强经济实力、较好产业基础、较大发展潜力，能带动周边区域、促进乡村振兴的重点乡镇。

（三）着力完善城乡基础设施。开展第三次全国土地调查，完成新一轮城市总规编制，启动全市国土空间规划、长株潭都市区总体规划和轨道交通网规划编制，积极推进“多规合一”。围绕长株潭一体化“三干两轨四连线”建设，有序实施芙蓉大道、潭州大道快速化改造，基本完成长潭城际轨道交通西线前期工作，协调长沙、株洲加快拉通滨江大道、昭山大道连接线。贯通湘江两岸防洪带，推进杨梅洲大桥、下摄司大桥和河东地区滨江路改造连通工程建设。加快荷塘综合客运枢纽与河西汽车总站建设，完善城际公交体系，逐步实现长株潭“一卡通”全覆盖。启动长株潭全国区域性物流枢纽工程建设，加快创建全国综合运输服务示范城市。加快醴娄高速湘潭段建设，建成岳临高速青山桥连接线，推动G320绕城线、西二环、伏林大道、莲花线、湘乡至韶山城际干线等项目建设。推进水上应急救援基地和湘潭港公共锚地等水运基础设施建设。加快万楼、碧泉湖、竹埠港、九华湖等重点片区开发。推进“千里湘江第一湾”建设，打造万楼、观湘门等景区景点。强化土地节约集约利用和批后监管，进一步规范土地市场秩序。创新投融资机制与建设安置模式，完成棚户区改造4000户。以城市“双修”为总揽，推进城市高质量发展。加快雨污分流改造，严格实行排污管网接入许可制度，启动河东、河西污水处理厂扩建工程。实施城市“微改造”计划，逐步畅通支路、街巷“微循环”。启动电网建设三年行动计划，再造一个全国一流的新湘潭电网。巩固“创文”成果，全力推进“创卫”，推动《湘潭市爱国卫生条例》立法。推进“绿心”生态修复和城市廊道建设，让森林走进城市，让城市拥抱森林，不断提升森林城市的生态魅力和亲和力。

（四）着力加强生态文明建设。深入推进污染防治攻坚战三年行动计划。全力打好蓝天保卫战，继续开展“飓风行动”“亮剑行动”“利剑行动”，开展柴油车尾气整治专项行动，推广餐饮油烟净化设备覆盖，严控工地扬尘和秸秆焚烧污染，推进湘钢大气污染防治计划，加快烟气脱硫脱硝等项目建设。健全空气质量监控网络，确保全年空气优良率不低于省定目标。深化湘江保护治理“一号工程”，加快港口和船舶污染物接收运转及处置设施建设，继续推进涟水流域污染专项整治和“一江两水”畜禽养殖污染整治，继续推进黑臭水体治理，构建长效管理机制。打造河长制升级版，建设功能更加齐全的监测和举报平台。加快土壤和固体废物污染治理，推进谭家山矿区生态修复工程，完成南天化工、锰矿四期、电化原址、湖铁地下水一期等重点治理项目。严厉打击非法采砂采矿，规划建设一批绿色矿山。推进垃圾焚烧发电厂、建筑垃圾消纳场等重点环保项目建设，实现餐厨垃圾无害化处理，完善乡镇垃圾运转体系，引入专业机构管理维护乡镇环保设施。设立绿色生态产业发展基金，鼓励新能源汽车发展，推广绿色建筑、装配式建筑。推动昭山建设省级绿色生态城区。建立健全长株潭统一的生态补偿机制和企业排污权交易制度。全面完成第二次全国污染源普查工作，严格落实领导干部离任自然资源资产审计。严禁生态环保领域出现“一刀切”问题。支持环保等公益诉讼。大力推进环保习惯养成和绿色文化宣传，让生态文明思想深入人心，使保护环境成为全市人民的自觉行动。

（五）着力推进乡村振兴战略。推进农业供给侧结构性改革，调优种养结构和区域布局，做优做响湘莲、槟榔、沙子岭猪等特色农产品品牌，做大做强农业龙头企业。落实粮食安全责任制，完成省定种植结构调整任务，推进休耕试点和安全利用。做好农村土地“三权分置”和集体产权制度改革，壮大农村集体经济，保障农民财产权益。抓好梅林桥、东郊、姜畲、银田、荷塘等现代农业示范园建设。推进农村人居环境整治，打好乡村振兴首仗，实现村庄规划全覆盖，强化农村房屋风貌管控，继续推进破败“空心房”和农村违法建筑治理，统筹开展农村生活垃圾治理、生活污水治理及厕所革命，深入开展“大棚房”问题专项整治。加快特色小镇建设，着力打造连片样板镇村。完成防洪薄弱环节治理、农村饮水安全等各类水利工程2万处，编制韶山灌区建设规划，持续推动“四好农村路”建设，完善供水供电、通信物流等农业农村配套设施，在韶山市建设农村人防疏散示范点。推进农膜回收和化肥农药使用量零增长行动，出台支持秸秆、畜禽废弃物资源化利用的政策措施。深入推进农业“双新双创”“三乡”工程和“绿领”培养计划，壮大新型职业农民队伍。鼓励乡村民宿发展，因地制宜发展农家游、休闲游。整合涉农资金，稳固农村金融，拓宽农业保险范围。打好非洲猪瘟防控阻击战，提高动物防疫应急处置能力。加强基层组织阵地建设，全面完成村级组织活动场所规范化建设任务。发展智慧农业，推进数字乡村建设，完善农村公共服务，加强乡风文明建设。坚决打赢脱贫攻坚战，推进未脱贫建档立卡贫困户精准帮扶工作，完成年度减贫任务。突出产业就业扶贫和社会救助兜底保障，持续开展“千企联万户”和脱贫攻坚“三落实”行动，巩固提升脱贫成果。

（六）着力释放改革发展活力。以供给侧结构性改革为主线，按照“巩固、增强、提升、畅通”方针，更多运用市场化法治化手段，加快新旧动能接续转换。持续深化“放管服”改革，全面实施招商引资重大产业项目投资企业承诺制，在“最多跑一次”的基础上推进“不见面审批”。健全政府购买服务绩效第三方评估机制，建立统一开放、竞争有序的市场体系。以培育市场化经营主体为导向，深化市属国有企业改革，完成地产集团市场化转型，增强城发、产业、交发集团市场竞争力，支持电化集团打造百亿级企业。实施优秀企业家成长计划，激发和保护企业家精神。落实中央、省委支持民营企业高质量发展系列政策，进一步鼓励、支持、引导民营企业做优做强，参照重大产业项目投资企业承诺制，办理民营企业新建项目和改扩建项目审批手续。依法打击破坏市场经济秩序、侵害企业家合法权益的违法犯罪行为。推进信用记录在政务领域的应用，全面开展守信激励和失信惩戒工作，牵头推动长株潭三市信用记录名单互认。继续深化金融改革，有效缓解企业“融资难、融资贵”和“融资意愿低”等问题。深化价格机制改革，放开一批政府定价的商品和服务价格。动态调整和完善涉企收费清单，推广“一表制”公示、“一站式”受理、“一票制”收费。基本完成长株潭两型试验区各项改革目标任务。加强国防动员和后备力量建设，积极推进省军区正规化建设试点。全面落实中央、省委和市委各项改革部署，让改革激发湘潭高质量发展的奋进力量。

（七）着力推动全方位对外开放。始终把招商引资作为经济发展的“第一菜单”，推行结对子、孵化式、产业链等精准招商新模式，进一步发挥好招商“小分队”、驻外办事处、异地商会、产业联盟成员企业等资源的积极作用。坚持把本籍客商作为倚重的力量，继续实施“对接湘商会、建设新家乡”活动，着力推动潭商总部回归、产业回归、资本回归和人才回归。积极探索和推进政府产业基金引导、片区整体开发等招商模式，舍得把最优的资源、最好的项目、最佳的地段拿出来招商。紧盯“三类500强”，主攻“长三角”“珠三角”“京津冀”等重点区域，积极参与湖南—粤港澳大湾区经贸活动周、湘商大会、京湘产业对接会等重大经贸活动，开展粤港澳大湾区产业招商主题年活动，精心组织“一谷三城”专场招商。成立推进招商引资重大项目落地实施和跟踪服务小组，着力解决“落地难”问题。鼓励企业积极融入“一带一路”建设，全面加强国际产能合作、劳务合作。支持企业搭乘中非经贸博览会、中国进口博览会、亚欧博览会、汉诺威工业展等会展平台，重点推介一批优质项目，促成一批战略合作，开拓一批重点市场。发挥好湘潭综保区作用，进一步做强开放平台。完善开放型经济考评考核机制，确保政策措施逐项落实、逐条兑现，奋力开创全面开放新格局，努力建设湖南对外开放新高地。

（八）着力保障和改善民生。办好新增城镇就业、农村危房改造等10件民生实事。实施就业优先、创业带动就业和职业技能提升等行动计划，健全覆盖全体劳动者的职业培训体系，推行终身职业培训制度，重点促进高校毕业生、返乡农民工和产能过剩企业职工等群体就业创业。实施莲城人才行动计划，确保驻潭高校毕业生留潭新增7000人以上。抓好农民工权益保障，推进农民工享受基本公共服务均等化，将符合条件的外来务工人员纳入住房租赁补贴保障。稳控住房价格，加大住房公积金制度覆盖面，推动完善住房市场体系和保障体系。深化社区网格化管理，发挥好社区楼栋长作用，推动住宅小区物业管理全覆盖，鼓励有条件的老旧小区增设电梯，加快高品质社区建设。突出建立共建、共治、共享社会治理新格局，推进信访法治化建设和信访“三无”乡村创建，全面推进社区矫正，加快市强制戒毒所扩建项目建设。继续推进公安派出所建设三年行动计划，深化“一村一辅警”“莲城快警”建设，实现城区接处警极速响应。推动扫黑除恶专项斗争向纵深发展，创建安定和谐的社会环境。加强防范和打击黄赌毒、电信诈骗、非法集资、传销等人民群众深恶痛绝的违法犯罪行为。深入开展创建国家安全发展示范城市，坚持不懈抓好专项整治和打非治违，全面推进“双预防”机制建设，坚决杜绝重特大安全生产事故。加强消防救援能力建设，努力开创应急管理新局面。深入开展双拥工作，争创新一轮全省双拥模范城。加强退役军人事务管理保障，维护军人家属合法权益。继续加大建设领域历史遗留问题处理。统筹抓好食品安全、防灾减灾、交通出行、殡葬惠民、扶残助残等各项民生工作。对于人民群众的身边事，我们将一年接着一年干，一件接着一件办，干就干实，办就办好，努力让人民群众有更多的获得感、安全感、幸福感。

（九）着力发展社会事业。办好人民满意的教育，优化教育设施布局，新建和实施岳塘湖湘学校、长郡韶山学校等一批教育重点项目，力争和平小学科大校区、富力雅礼中学、昭山华鑫高级中学等建成开学，全力推进化解大班额。加强教师队伍建设，加快学前教育改革发展，加大新建公办幼儿园力度，推动全国义务教育优质均衡发展县创建。综合防控儿童青少年近视，完善中小学课后服务机制，着力解决人民群众关心的“三点半”等教育领域热点难点问题。抓好新高考改革，推动特色高中普及发展。推进职教体系建设，大力推进现代学徒制、工学结合和集团化办学模式改革。继续支持驻潭高校“双一流”建设、校园及周边环境整治、服务配套设施建设等工作，推进软件学院专升本，实现北津学院建成开学。推进长株潭职业教育实习实训基地共建共享，实现三市院校毕业生就业信息网络互联。建设健康湘潭。逐步提高基本医保和大病保险保障水平，严格落实基本养老金和居民医保补助标准提标等相关政策。全面推进县乡村医疗卫生机构一体化，提升基层医疗卫生服务能力。启动九华医院建设。推进“智慧医疗健康”建设，切实改善医疗服务，有效提升临床重点专科能力，传承发展中医药事业。完成长株潭血液中心采供血平台建设，实现库存信息共享和用血费用异地报销。推进长株潭医疗急救平台和急救指挥一体化系统建设。加快推进居家和社区养老服务改革试点，打造医养结合湘潭品牌。深化全国社保卡综合应用试点，推动社保卡“一卡通”。启动社会救助综合配套改革，构建新型社会救助体系，健全农村留守儿童、残疾人关爱保护体系，建立残疾儿童康复救助制度，建设雨湖福利中心。完善基层文化设施，确保村级综合性文化服务中心建成率达80%以上。广泛开展群众文体活动，推动竞技体育快速发展。加强文艺创作，强化文化市场监管。加强对非物质文化遗产资源的保护开发，支持韶山灌区、云门寺、陈赓故居申报全国重点文物保护单位。推进昭山文化产业园创建国家级文化产业示范园。加强湖湘文化、红色文化等各类文化资源的传承与发展，努力打造具有独特魅力、充沛活力的“文化创意产业城”。

（十）着力加强政府自身建设。始终把政治建设摆在首位，牢固树立“四个意识”，坚定“四个自信”，践行“两个维护”。坚决贯彻党中央大政方针，认真落实省委、市委决策部署。推进法治政府建设。严格执行重大行政决策法定程序，以依法行政带动全社会尊法守法。按照机构改革总体部署，加快构建系统完备、科学规范、运行高效的政府机构职能体系。自觉接受人大及其常委会法律监督、工作监督和政协民主监督，主动接受司法监督、社会监督和舆论监督，认真听取各民主党派、工商联、无党派人士、新社会阶层人士和人民团体意见，高质量办好人大代表建议和政协提案。推进廉洁政府建设。主动接受监察监督，强化审计监督，深化廉政风险防控，严管财政资金使用、公共资源交易、国有资产管理等重点领域和关键环节。自觉扛起全面从严治党主体责任和“一岗双责”，严格落实中央八项规定及实施细则精神，驰而不息纠“四风”，始终做到心中有戒、行之有界。坚持打“紧算盘”，确保“三公”经费再压减，严控公务出国、公务用车、公务接待开支。推进实干政府建设。多倾听基层干部的心声，多关爱基层干部的成长与生活，多为基层干部鼓鼓掌、暖暖心、充充电。切实整治“痕迹管理”、陪同陪会、文件简报和检查考核过多过滥过度问题，让干部集中精力抓落实，一门心思求发展。不采华名，不兴伪事，对认准的事决不优柔寡断，对决定的事决不拖沓观望，对铺开的事决不半途而废，真正把蓝图上绘的、文件上写的、会议上说的，落实为实实在在的行动和效果。推进担当政府建设。带领政府一班人和全体公务员，不忘初心，不离本心，多钻“矛盾窝”，多喊“跟我来”，多啃“硬骨头”，为敢想的人“鼓足劲”，为敢干的人“加满油”，为敢试的人“卸包袱”，激发积极作为、主动履责的动力，锤炼敢于担当、勇毅笃行的政风，增强“不为风险所惧、不为干扰所惑”的勇气，拿出“想尽一切办法、穷尽一切可能”的韧劲，把一代又一代湘潭人孜孜以求、不懈奋斗的伟大事业推向前进！

各位代表，我们都是追梦人，我们都在努力奔跑。让我们高举习近平新时代中国特色社会主义思想伟大旗帜，更加紧密地团结在以习近平同志为核心的党中央周围，在省委、省政府和市委的坚强领导下，上下同欲，精进前行，以优异的成绩迎接中华人民共和国成立70周年！
